# Fetal diagnosis of meconium periorchitis: A case report

**DOI:** 10.1002/ccr3.8319

**Published:** 2023-12-15

**Authors:** Ahmed S. Z. Moustafa, Sarah Araji

**Affiliations:** ^1^ Department of Obstetrics and Gynecology, Division of Maternal Fetal Medicine University of Mississippi Medical Center Jackson Mississippi USA

**Keywords:** bowel perforation, echogenic fetal scrotum, enlarged fetal scrotum, neonatal hydrocele, prenatal ultrasound

## Abstract

Fetal meconium periorchitis (MPO) is rare prenatal diagnosis associated with meconium peritonitis. The prenatal ultrasound finding consists of an enlarged fetal scrotum with echogenic fluid and debris. In this report, we describe a case in which a prenatal diagnosis of MPO was accurately made at 32 weeks of gestation. The neonate delivered without complications, underwent immediate evaluation followed by major surgery, and ultimately had a favorable outcome. An accurate prenatal diagnosis is important to counsel the patient in a multidisciplinary approach. This case highlights the prenatal ultrasound findings as well as the neonatal presentation and the possibility for conservative management by pediatric urology.

## Graphical abstract

Ultrasound image of an enlarged fetal scrotum in axial plane.

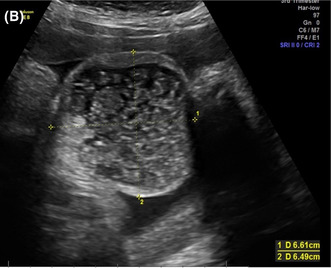

## 1 INTRODUCTION

Fetal meconium periorchitis (MPO) is an uncommon finding in utero; the most common presentation is an enlarged scrotum with echogenic debris seen on ultrasound.^1^ MPO can be secondary to fetal bowel perforation secondary to thick meconium which can be seen in cystic fibrosis.^2^ Other causes of bowel perforation are bowel anomalies and an ischemic event.^3^

Recognizing this rare diagnosis prenatally is crucial for appropriate care coordination and planned delivery at an institution with access to neonatology, pediatric surgery, and urology services to prevent delay in postnatal diagnosis as well as unnecessary interventions.

## 2 CASE REPORT

### 2.1 Prenatal course

**FIGURE 1:**
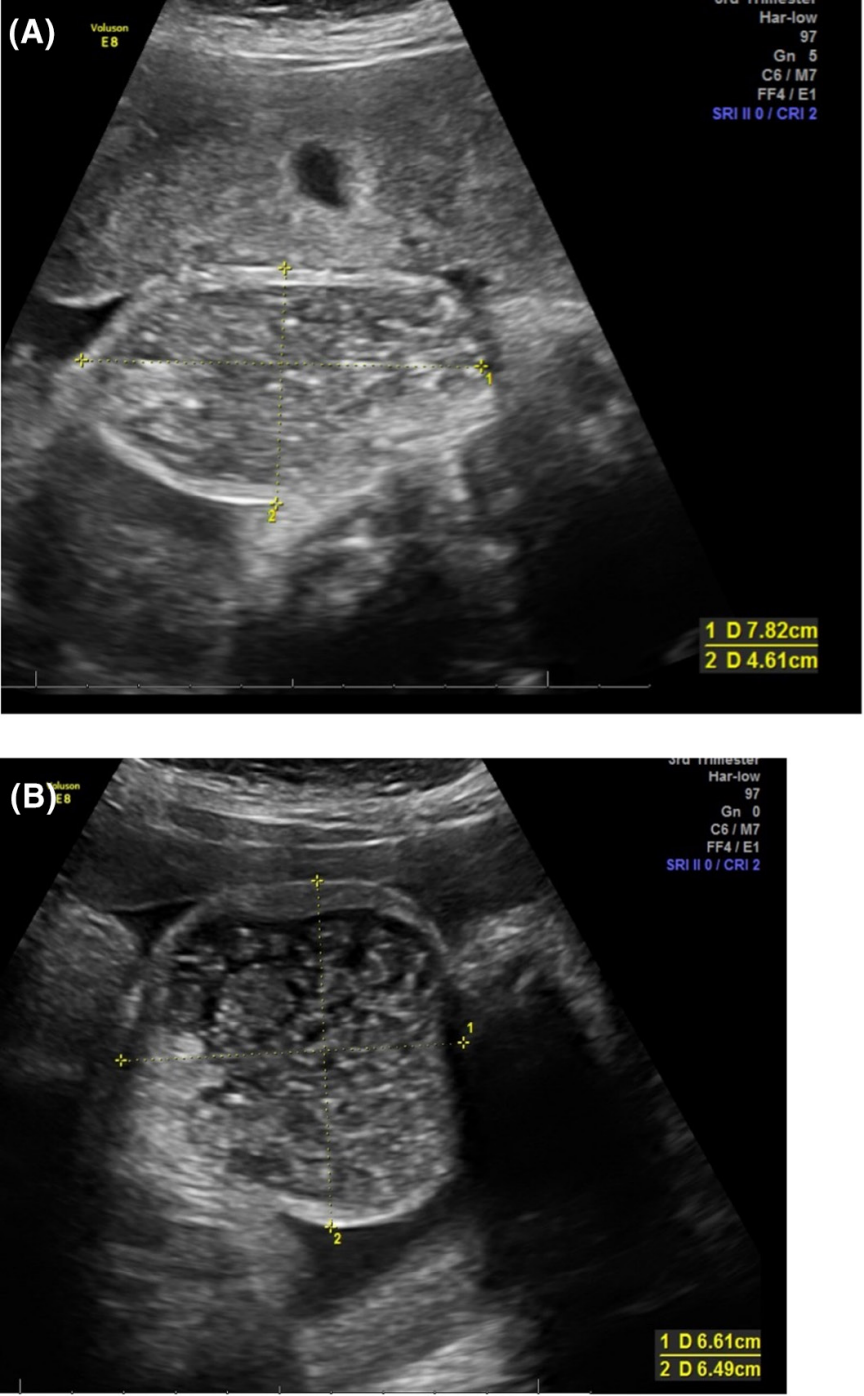
(A, B) Ultrasound images of an enlarged fetal scrotum (A: coronal plane, B: axial plane) filled with echogenic fluid, debris, and calcifications.

**FIGURE 2:**
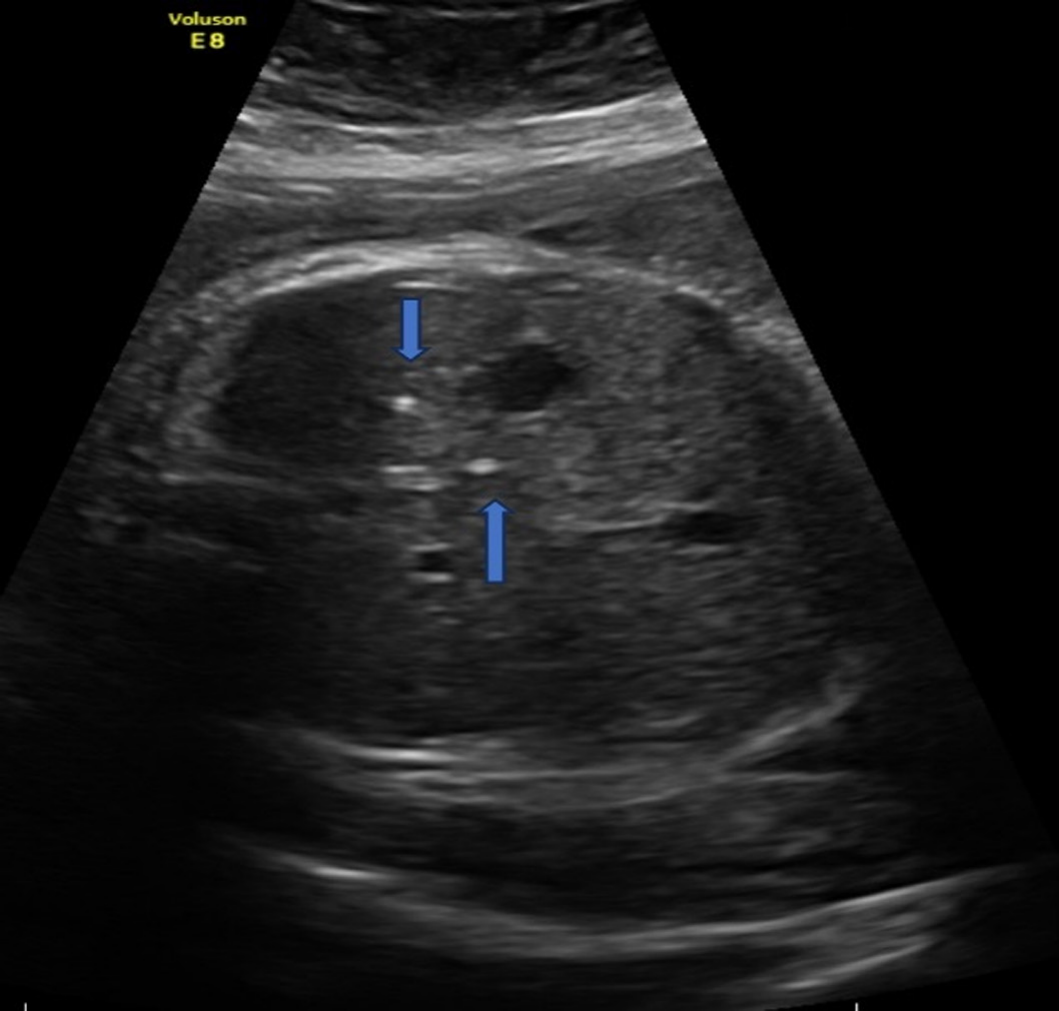
Ultrasound axial image of the fetal abdomen showing liver calcifications (Blue arrows).

A 35‐year‐old woman, gravida 1 para 0, was referred to our fetal center at 32 weeks of gestation for evaluation of an enlarged fetal scrotum. On ultrasound, an enlarged fetal scrotum, with a large volume of echogenic fluid, debris, and calcifications was seen (Figure 1). Testicular decent was noted (Figure 1). After careful evaluation of the fetal abdomen liver calcifications were also noted (Figure 2). There was no evidence of fetal ascites, dilated bowel loops, or meconium pseudocysts. Maternal prenatal genetic carrier screening was negative for cystic fibrosis. Testing for cytomegalovirus (CMV) and other infectious etiologies using maternal serology was also negative. Neonatology, pediatric surgery, and pediatric urology were consulted prenatally for a suspected diagnosis of MPO. Induction of labor was scheduled at 37 weeks of gestation secondary to gestational hypertension. A 3210‐g male neonate was delivered vaginally with no complications.

### 2.2 Neonatal course

**FIGURE 3:**
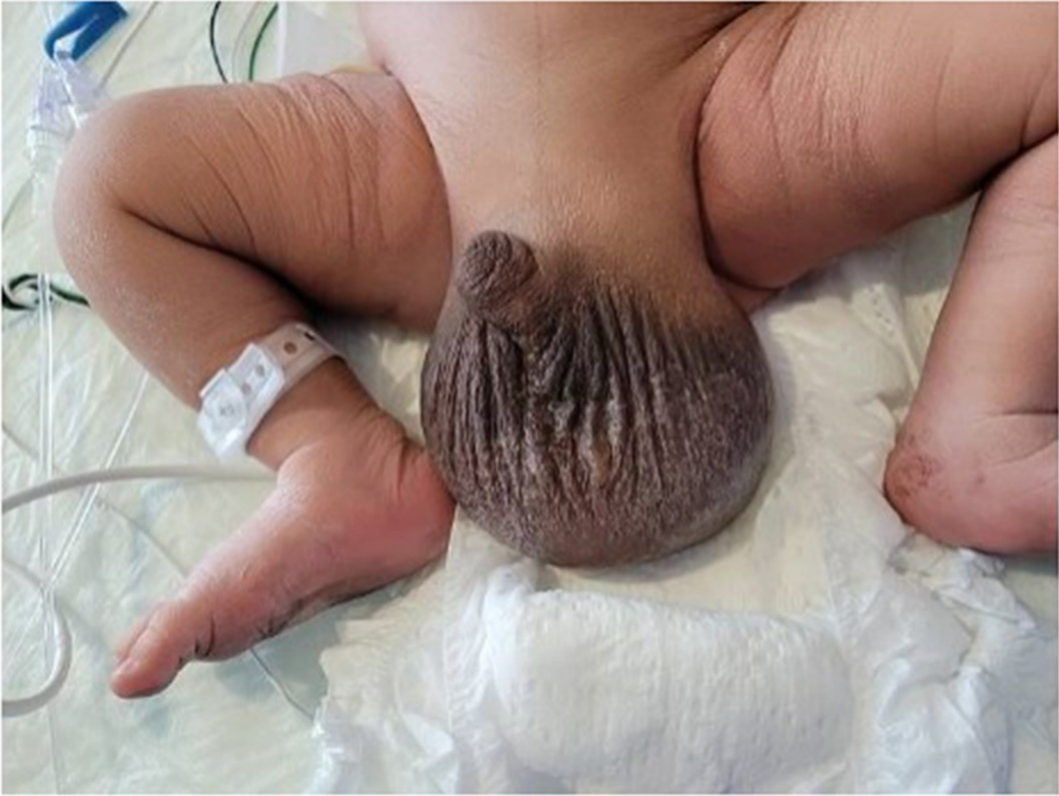
Enlarged scrotum of neonate at day of life zero.

A large scrotum was noted upon physical examination (Figure 3), and postnatal testicular ultrasound performed on day of life zero (DOL) was consistent with meconium periorchitis. Abdominal X‐ray noted large loculated appearing pneumoperitoneum collection over the central upper abdomen. Exploratory laparotomy was performed on DOL 1, meconium and meconium pseudocyst were noted without overt bowel perforation. Small bowel resection was performed with ileostomy secondary to extensive adhesions. The bilateral inguinal rings were inspected and noted to be plugged with meconium. On DOL 42, ileostomy reversal was performed with resection of 10 cm of terminal Ileum and right colon after identifying perforation sites in these bowel segments, followed by side‐by‐side ileocolic anastomosis. Urology was consulted and recommended conservative management with follow‐up showing improvement in meconium hydroceles.

## 3 DISCUSSION

Fetal meconium periorchitis is a rare complication that occurs secondary to meconium peritonitis.^2^ Fetal bowel perforation leads to leakage of sterile meconium into the abdominal cavity causing meconium peritonitis, this leads to passage of meconium through a patent processus vaginalis resulting in an inflammatory reaction of the soft tissue around the testicles and MPO.^4^ Fetal bowel perforation can be caused by a primary ischemic event, a bowel anomaly such as atresia or volvulus.^3^ In cases of suspected meconium peritonitis, testing for cystic fibrosis is recommended as it accounts for up to 40% of cases.^5^, ^6^ This is secondary to the abnormal cystic fibrosis transmembrane conductance regulator (CFTR) resulting in thick mucus. As a result, viscid meconium is formed and can cause physical obstruction of the terminal ileum.^7^ Prenatal diagnosis is challenging, and, therefore, MPO is rarely accurately diagnosed in utero,^2^ it is often misdiagnosed as a hydrocele, inguinal hernia, hematoma, or testicular tumor. The diagnosis is commonly made in the first few months after birth; however, delayed diagnosis for up to 5 years of life has been reported.^8^ Neonates usually present with a scrotal swelling, a large hydrocele, or a scrotal mass palpated on physical exam.^9^ In some cases, no abnormality is identified at time of birth.^2^ Ultrasound findings suggesting fetal MPO include enlarged scrotum, cystic, or solid mass, simple, or complex calcifications that may cast acoustic shadows. Furthermore, findings supporting a diagnosis of meconium peritonitis include dilated bowel, intraperitoneal calcifications, and meconium pseudocysts. It is important to note that leakage of bowel contents overtime could lead to formation of a pseudocyst as a fibrous wall is formed around the spilled meconium,^5^ in a case series and meta‐analysis that examined prenatal ultrasound findings in 244 cases with a diagnosis of meconium peritonitis, presence of meconium pseudocysts was the strongest predictor for the need of postnatal surgical management.^10^ However, as in the described patient, meconium pseudocysts are not always visualized prenatally. In a retrospective study that included 37 cases of meconium peritonitis, pseudocysts were only identified prenatally in 2 cases.^11^ It is crucial to look for small bowel loops with peristaltic waves within the scrotum as this would be suggestive of an inguinoscrotal hernia rather than meconium periorchitis.^12^ MPO diagnosed in the prenatal period tends to have a good prognosis as intestinal perforation usually will heal before delivery.^13^ More importantly the mortality rate after meconium peritonitis has decreased significantly secondary to improved fetal diagnostic tools and management.^14^ The management of MPO includes conservative and surgical approaches.

In cases where bowel perforation does not heal neonates may develop bowel distention and an acute abdomen requiring immediate surgery.^15^ In our case, the neonate needed surgical intervention on DOL 1 for small bowel resection after suspected pneumoperitoneum, whereas the pediatric urology team followed a conservative approach to manage the enlarged scrotum. In conclusion, a multidisciplinary team (MDT) is needed for counseling, prompt intervention and to avoid unnecessary surgery as orchidectomy has been reported in a benign case of meconium orchitis due to a concern for a rhabdomyosarcoma.^16^ Our case emphasizes the importance of recognizing these cases antenatally to coordinate delivery at a tertiary care center with a MTD approach including Maternal‐Fetal Medicine, neonatology, pediatric urology, and pediatric surgery.

## AUTHOR CONTRIBUTIONS

**Ahmed S. Z. Moustafa:** Conceptualization; writing – original draft; writing – review and editing. **Sarah Araji:** Conceptualization; writing – original draft; writing – review and editing.

## CONFLICT OF INTEREST STATEMENT

The authors declare no conflict of interest.

## FUNDING INFORMATION

No funding to declare.

## CONSENT

Written informed consent was obtained from the patient's parent to publish this report in accordance with the journal's patient consent policy.

## Data Availability

Data sharing is not applicable to this article as no new data were created or analyzed in this study.

## References

[1] Alanbuki AH , Bandi A , Blackford N . Meconium periorchitis: a case report and literature review. Can Urol Assoc J. 2013;7(7–8):E495‐E498. 23914267 10.5489/cuaj.316 PMC3713149

[2] Regev RH , Markovich O , Arnon S , Bauer S , Dolfin T , Litmanovitz I . Meconium periorchitis: intrauterine diagnosis and neonatal outcome: case reports and review of the literature. J Perinatol. 2009;29(8):585‐587. 19638993 10.1038/jp.2009.15

[3] Boccon‐Gibod L , Roucayrol AM . Meconium periorchitis. Pediatr Pathol. 1992;12(6):851‐856. 1448393 10.3109/15513819209024243

[4] Gililland A , Carlan SJ , Greenbaum LD , Levy MC , Rich MA . Undescended testicle and a meconium‐filled hemiscrotum: prenatal ultrasound appearance. Ultrasound Obstet Gynecol. 2002;20(2):200‐202. 12153675 10.1046/j.1469-0705.2002.00663.x

[5] Reynolds E , Douglass B , Bleacher J . Meconium peritonitis. J Perinatol. 2000;20(3):193‐195. 10802847 10.1038/sj.jp.7200287

[6] Casaccia G , Trucchi A , Nahom A , et al. The impact of cystic fibrosis on neonatal intestinal obstruction: the need for prenatal/neonatal screening. Pediatr Surg Int. 2003;19(1–2):75‐78. 12721730 10.1007/s00383-002-0781-8

[7] Sathe M , Houwen R . Meconium ileus in cystic fibrosis. J Cyst Fibros. 2017;16(Suppl 2):S32‐S39. 28986020 10.1016/j.jcf.2017.06.007

[8] Mene M , Rosenberg HK , Ginsberg PC . Meconium periorchitis presenting as scrotal nodules in a five year old boy. J Ultrasound Med. 1994;13(6):491‐494. 8083953 10.7863/jum.1994.13.6.491

[9] Wax JR , Pinette MG , Cartin A , Blackstone J . Prenatal sonographic diagnosis of meconium periorchitis. J Ultrasound Med. 2007;26(3):415‐417. 17324997 10.7863/jum.2007.26.3.415

[10] Shinar S , Agrawal S , Ryu M , et al. Fetal meconium peritonitis ‐ prenatal findings and postnatal outcome: a case series, systematic review, and meta‐analysis. Ultraschall Med. 2022;43(2):194‐203. 32575129 10.1055/a-1194-4363

[11] Chen CW , Peng CC , Hsu CH , et al. Value of prenatal diagnosis of meconium peritonitis: comparison of outcomes of prenatal and postnatal diagnosis. Medicine (Baltimore). 2019;98(39):e17079. 31574807 10.1097/MD.0000000000017079 PMC6775423

[12] Frati A , Ducarme G , Vuillard E , et al. Prenatal evaluation of a scrotal mass using a high‐frequency probe in the diagnosis of inguinoscrotal hernia. Ultrasound Obstet Gynecol. 2008;32(7):949‐950. 19009574 10.1002/uog.6241

[13] Jimenez‐Cabanillas MV , Vetter‐Laracy S , Cobo P , Roca Jaume A , Maroto‐Garcia CM , Moreno MA . Meconial hydrocele as first sign of acute intestinal perforation in a preterm baby. Arch Dis Child Fetal Neonatal ed. 2019;104(2):F169‐F170. 30269084 10.1136/archdischild-2018-315737

[14] Tseng JJ , Chou MM , Ho ES . Meconium peritonitis in utero: prenatal sonographic findings and clinical implications. J Chin Med Assoc. 2003;66(6):355‐359. 12889504

[15] Eckoldt F , Heling KS , Woderich R , Kraft S , Bollmann R , Mau H . Meconium peritonitis and pseudo‐cyst formation: prenatal diagnosis and post‐natal course. Prenat Diagn. 2003;23(11):904‐908. 14634976 10.1002/pd.720

[16] Williams HJ , Abernethy LJ , Losty PD , Kotiloglu E . Meconium periorchitis—a rare cause of a paratesticular mass. Pediatr Radiol. 2004;34(5):421‐423. 14685788 10.1007/s00247-003-1079-2

